# Special Issue “Dynamics and Processes at Laser-Irradiated Surfaces—A Themed Issue in Honor of the 70th Birthday of Professor Jürgen Reif”

**DOI:** 10.3390/nano13030611

**Published:** 2023-02-03

**Authors:** Florenta Costache, Stéphane Valette, Jörn Bonse

**Affiliations:** 1 Fraunhofer Institute for Photonic Microsystems, IPMS, Maria-Reiche-Str. 2, 01109 Dresden, Germany; 2 Ecole Centrale de Lyon, LTDS, 36 Avenue Guy de Collongue, 69134 Ecully, France; 3 Bundesanstalt für Materialforschung und -prüfung (BAM), Unter den Eichen 87, 12205 Berlin, Germany

The Special Issue “Dynamics and Processes at Laser-irradiated Surfaces” is dedicated to the 70th birthday of Jürgen Reif, retired full professor, former Chair of Experimental Physics II of the Faculty of Physics of the Brandenburg University of Technology Cottbus—Senftenberg in Germany.

This Special Issue was organized by one of his former PhD students and collaborators (F.C.), one of his collaborators (S.V.), and one of his external colleagues (J.B.) in recognition of Jürgen Reif’s long-lasting scientific contributions and research lines in the fields of photonics, semiconductor technology, optical spectroscopy, surface dynamics, in situ measurement techniques, experimental and theoretical investigations of laser–matter interaction, applications of surface functionalization through laser-induced micro- and nanostructures, laser processing of polymers, numerical modeling of surface processes, etc.

Because these scientific areas lie at the boundaries between nonlinear optics and spectroscopy, surface science, solid-state physics, materials science and nanotechnology, and in honor and recognition of Prof. Reif’s scientific contributions, this Special Issue was simultaneously organized in the two journals *Molecules* and *Nanomaterials*, published in a separate branch for each journal [1,2].

For more than 25 years, apart from educating and qualifying university students for the future, the work of Prof. Reif has been very actively devoted to the study of fundamental mechanisms, dynamics, and applications of nano- and micrometer-scale laser-induced periodic surface structures (LIPSS), with a special focus on self-organization processes [3]. His contributions have revived and strongly stimulated the competition of the two main approaches (“schools”) to explain LIPSS, either via matter reorganization or via coherent electromagnetic scattering effects, and investigated their dynamics and applications in the fields of surface wetting and tribology [3,4]. Jürgen Reif and his research group very actively promoted research in the direction of self-organization and made important contributions to this subject [5,6,7,8]. During the last few years and with the development and numerical implementation of advanced multi-scale models involving various physical effects, the two LIPSS schools are merging now [4,9,10], while new applications of LIPSS are currently systematically being screened and explored [11,12,13]. LIPSS represent an important cornerstone for ultrafast laser nanostructuring and the pursuit to extreme scales [14]. This is also reflected by numerous LIPSS-related papers contained in this collection of articles.

Our Special Issue attracted high-quality contributions from both academics and from industry and finally bundles together 1 review paper of Jürgen Reif [3], 1 perspective article [4], and 17 original research articles [15,16,17,18,19,20,21,22,23,24,25,26,27,28,29,30,31]. These publications are focusing on the latest achievements in areas of laser–matter interaction [16,18,22,23,27,30,31], nonlinear optics and photonics [22,23,26], spatial beam shaping [15,28], surface dynamics [4,17,22,23], micro- and nanotechnology [16,18,20,24,26,27,31], laser-induced periodic surface structures (LIPSS) [3,4,19,20,21,27,28,31], surface functionalization and applications of laser-textured surfaces [3,4,15,17,21,24,25,27,29,30], and surface wetting and tribology [15,17,25,29]. All of them represent a rewarding dedication to the scientific field and the contributions of Prof. Jürgen Reif, honoring his 70th birthday.

Finally, the Guest Editors would like to express their sincere gratitude to all authors and reviewers of this Special Issue for their contribution to this Special Issue and to the editorial staff of *Nanomaterials* and *Molecules* for their professional support and guidance.

## Data Availability

Not applicable.
